# Soluble Endoglin, Transforming Growth Factor-Beta 1 and Soluble Tumor Necrosis Factor Alpha Receptors in Different Clinical Manifestations of Preeclampsia

**DOI:** 10.1371/journal.pone.0097632

**Published:** 2014-05-22

**Authors:** Luiza O. Perucci, Karina B. Gomes, Letícia G. Freitas, Lara C. Godoi, Patrícia N. Alpoim, Melina B. Pinheiro, Aline S. Miranda, Antônio L. Teixeira, Luci M. Dusse, Lirlândia P. Sousa

**Affiliations:** 1 Departamento de Análises Clínicas e Toxicológicas, Faculdade de Farmácia, Universidade Federal de Minas Gerais, Belo Horizonte, Minas Gerais, Brazil; 2 Programa de Pós-Graduação em Análises Clínicas e Toxicológicas, Faculdade de Farmácia, Universidade Federal de Minas Gerais, Belo Horizonte, Minas Gerais, Brazil; 3 Programa de Pós-Graduação em Ciências Farmacêuticas, Faculdade de Farmácia, Universidade Federal de Minas Gerais, Belo Horizonte, Minas Gerais, Brazil; 4 Faculdade de Medicina, Universidade Federal de São João Del Rei, São João Del Rei, Minas Gerais, Brazil; 5 Laboratório Interdisciplinar de Investigação Médica, Faculdade de Medicina, Universidade Federal de Minas Gerais, Belo Horizonte, Brasil; Medical Faculty, Otto-von-Guericke University Magdeburg, Medical Faculty, Germany

## Abstract

**Background:**

Despite intensive research, the etiopathogenesis of preeclampsia (PE) remains uncertain. Inflammatory and angiogenic factors are thought to play considerable roles in this disease. The objective of this study was to investigate the association between soluble endoglin (sEng), transforming growth factor beta-1 (TGF-β1) and tumor necrosis factor alpha soluble receptors (sTNF-Rs) and the clinical manifestations of PE.

**Methods:**

Plasma levels of sEng, TGF-β1 and sTNF-Rs were determined by ELISA in 23 non-pregnant, 21 normotensive pregnant and 43 PE women. PE women were stratified into subgroups according to the severity [mild (n = 12) and severe (n = 31)] and onset-time of the disease [early (n = 19) and late (n = 24)].

**Results:**

Pregnancy was associated with higher levels of sEng, sTNF-R1 and sTNF-R2 than the non-pregnant state. Moreover, PE women had higher levels of sEng and sTNF-R1 than normotensive pregnant women. No difference was found in TGF-β1 levels, comparing the three study groups. Late PE had higher levels of sTNF-R1 and sTNF-R2 than early PE. No significant differences were found in sEng and TGF-β1 comparing early and late PE. sEng levels were higher in severe PE than in mild PE and no difference was found for TGF-β1, sTNF-R1 and sTNF-R2 levels. There was a positive correlation among sEng, TNF-R1 and sTNF-2 levels. Logistic regression analysis revealed that primiparity and sEng levels are independently associated with the development of PE. Furthermore, sEng levels are independently associated with the disease severity.

**Conclusions:**

These results suggest that pregnancy is a condition associated with higher levels of anti-angiogenic and pro-inflammatory factors than the non-pregnant state and that PE is associated with an imbalance of these factors in the maternal circulation.

## Introduction

Preeclampsia (PE) is a disorder of human pregnancy characterized by hypertension and proteinuria on or after the 20^th^ week of gestation. Traditionally, PE has been classified according to the severity of clinical signs and laboratory findings in mild and severe PE [1]. In recent years, the classification of PE according to gestational age (GA) at the onset of the disease has been also considered [2]. The early onset (GA < 34 weeks) is characterized by inadequate and incomplete trophoblast invasion of maternal spiral arteries, while the late onset (GA ≥34 weeks) is associated with normal fetal growth and no changes in uterine spiral arteries [3].

PE etiopathogenesis is associated with placental hypoxia and/or ischemia and excessive oxidative stress. Release of soluble factors from the ischemic placenta into maternal plasma plays a central role in the ensuing endothelial dysfunction, which is the most prominent feature of the disease [4]. Some candidates for these unknown ‘preeclampsia factors’ include pro-inflammatory cytokines, microparticles and anti-angiogenic factors [4], [5].

An imbalance between pro-angiogenic factors such as vascular endothelial growth factor (VEGF) and placental growth factor (PlGF), and anti-angiogenic factors such as soluble fms-like tyrosine kinase 1 (sFlt1) and soluble endoglin (sEng), has been observed before the onset of PE and after the clinical diagnosis [6]–[9].

Endoglin (Eng), or CD105, is a homodimeric transmembrane glycoprotein localized on cell surfaces that functions as a co-receptor for transforming growth factor (TGF)-β1 and TGF-β3 isoforms [10]. Soluble endoglin (65kDa) might play an anti-angiogenic effect in PE through binding to circulating TGF-β1, thus preventing its interaction with cell membrane, and consequently the pro-angiogenic and vasodilators effects of TGF-β1 in the normal endothelium [11]. Previous studies demonstrated the involvement of TGF-β1 in the pathophysiology of PE but the results are conflicting. Some studies have found higher levels of TGF-β1 in PE, while in others there was no difference between PE and normotensive pregnancies [12], [13].

It is well established that an excessive maternal systemic inflammatory response to pregnancy is also involved in the pathogenesis of PE [14], [15]. Our research group has previously reported higher levels of pro-inflammatory cytokines (IL-6, IL-8, IFN-γ and TNF-α), and decreased levels of the anti-inflammatory cytokine IL-10 in PE [16]. Other studies have also reported that maternal circulating concentration of TNF soluble receptors sTNF-R1 (55 kDa) and sTNF-R2 (75 kDa) are significantly increased in preeclamptic and normotensive pregnant women under risk to develop this disease [12], [17]–[19].

The aim of this study was to evaluate the sEng, TGF-β1, sTNF-R1 and sTNFR-2 levels in different forms of PE and to compare these variables to normotensive pregnant and healthy non-pregnant women, aiming to better understand the role of inflammation and endothelial dysfunction in PE pathogenesis. Our findings suggest that pregnancy is a condition associated with higher levels of anti-angiogenic and pro-inflammatory factors than the non-pregnant state and that PE is associated with an imbalance of these factors in the maternal circulation.

## Methods

### Study population

This case control study included 87 women: 23 non-pregnant, 21 normotensive pregnant and 43 preeclamptic. All participants were selected from public Brazilian institutions and provided written informed consent for the collection and use of samples for research purposes under the protocols approved by the Ethical Committee of Universidade Federal de Minas Gerais, Brazil (Institutional Review Board Project #0618.0.203.000-10).

Preeclamptic women were stratified into subgroups according to the severity [mild (n = 12) and severe (n = 31)] and to the onset of the disease [early-onset (n = 19) and late-onset (n = 24)]. All pregnant women were at the third trimester of gestation.

Severe PE was defined as blood pressure ≥160/110 mmHg and proteinuria ≥2 g/24hs or at least 3+ reading on dipstick in a random urine specimen after 20 weeks of pregnancy. Mild PE was defined as blood pressure ≥140/90 mmHg and proteinuria ≥300 mg/24hs or ≥1+ reading on dipstick in a random urine specimen after 20 weeks of pregnancy [1]. Early- and late-onset PE were classified according to gestational age of the onset of the disease (<34 or ≥34 weeks, respectively) [2]. Normotensive pregnant women had blood pressure ≤120/80 mmHg and no history of hypertension. Non-pregnant women included healthy premenopausal female.

Exclusion criteria common for the three groups were chronic hypertension, obesity, diabetes, cancer, homeostatic abnormalities, acute infectious disease, cardiovascular, autoimmune, renal and hepatic diseases.

### Blood sampling

The whole blood samples were collected into EDTA-K_3_ 1.8 mg/mL tubes and centrifuged at 2500 g for 20 min at room temperature to obtain the plasma samples. The aliquots were stored at −80°C until assayed.

### Enzyme-linked immunosorbent assay for plasma measurements

Plasma concentrations of sEng, TGF-β1, sTNF-R1 and sTNF-R2 were determined using commercially available kits purchased from R&D Systems (Minneapolis, MN). In general, plasma samples were diluted to 1 to 6 (for sEng), 1 to 2 (for TGF-β1) and 1 to 10 (for sTNF-R1 and sTNF-R2) and assayed according to the manufacturer's instructions. Samples from cases and controls were run simultaneously at the same plate and the assays were blind for patient identification and disease status. Absorbance was measured using a microplate reader and sample readings were extrapolated against a concurrently run standard curve. Concentrations were reported as nanograms per milliliter (ng/mL) for sEng and picograms per milliliter (pg/mL) for TGF-β1, sTNF-R1 and sTNF-R2. The intra- and inter-assay coefficients of variation were, respectively: sEng (2.8–3.2%; 6.3–6.7%), TGF-β1 (1.9–2.9% ; 6.4–9.3%), sTNF-R1 (3.6–5.0%; 3.7–8.8%) and sTNF-R2 (2.6–4.8%; 3.5–5.1%).

The Endoglin monoclonal antibody recognizes natural and recombinant human Eng. The following factors were assayed for cross-reactivity: recombinant human Activin A, Activin RIA, Activin RIIA, Activin RIIB, BMPR-IA, BMPR-IB, BMP-2, BMP-4, BMP-5, BMP-6, Follistatin 288, Follistatin 300, Follistatin 315, Inhibin A, Inhibin B, LAP, TGF-α, TGF-β1, TGF-β1.2, TGF-β2, TGF-β3, TGF-β RII and TGF-β RIII; recombinant mouse BMPR-IA and BMPR-IB; other recombinants: rat Agrin, porcine TGF-β2, amphibian TBF-β5; natural proteins: human TGF-β1 and porcine TGF-β1. No significant cross-reactivity or interference was observed.

The polyclonal antibody against TGF-β1 recognizes natural and recombinant TGF-β1. Besides those parameters tested for Eng antibody cross-reactivity, other factors were also tested for TGF-β1 antibody cross-reactivity, such as: recombinant human BMP-3, BMP-3b, BMP-8b, BMP-10, BMP-15, BMPR-II, and TGF-β RI; recombinant mouse BMP-3b and TGF-β RI; zebrafish BMP-2a and natural protein porcine TGF-β2. No significant cross-reactivity or interference was observed. Latent TGF-β1 complex has approximately 15% cross-reactivity in this assay. This assay also recognizes human TGF-β1.2 and porcine, mouse, rat, and canine TGF-β1. Significant interference was observed with rmTGF-β RII. However, no naturally occurring soluble mouse TGF-β RII has been reported to date [20], so this observed interference may be of no concern when measuring natural TGF-β1.

Recombinant human TNF-α, TNF-β and sTNF-R2, recombinant mouse TNF-α and sTNF-R1, porcine TNF-α and rat TNF-α were assayed for TNF-R1 antibody cross-reactivity. No significant cross-reactivity or interference was observed.

Recombinant human sTNF-R1 and TNF-β and recombinant mouse TNF-α, sTNF-R1 and sTNF-R2 were assayed for TNF-R2 antibody cross-reactivity. No significant cross-reactivity or interference was observed. Recombinant human TNF-α did not cross-react in this assay but did interfere at concentrations greater than 6.25 ng/mL.

### Other laboratory parameters

Aspartate aminotransferase (AST), alanine aminotransferase (ALT), lactate dehydrogenase (LDH) and creatinine plasma levels were also analyzed. The normal ranges considered in this study were: AST<31 U/L, ALT<34 U/L, LDH = 200–480 U/L and creatinine = 0.6–1.1 mg/dL [21]. The laboratory data were obtained from medical records at the time of sampling and were only available for PE women.

### Statistical analysis

Statistical analysis was performed with SPSS software version 13.0 (SPSS Inc., Chicago, IL, USA). The normality of continuous variables was assessed using Shapiro-Wilk's *W*-test. The comparison of continuous variables not normally distributed was made by Kruskal-Wallis test for three groups. When differences were detected among the groups, they were compared two by two by the Mann-Whitney *U*-test with *Bonferroni's* correction. The comparison of variables with normal distribution was performed by Student *T*-test (two groups) or ANOVA with *post-hoc* LSD test (three groups). The comparison of categorical variables was performed by Pearson chi-square (χ^2^) test. Parametric data are reported as mean ± standard deviation (SD), nonparametric data as median (25th–75th percentiles) and categorical variables as absolute number (percentage). Correlation was evaluated by Spearman coefficients (r_s_). The continuous and categorical variables that showed statistical significance among the studied groups were included in multivariate logistic regression models. Three models were developed considering as dependent variables: PE pregnancy x normotensive pregnancy, early PE x late PE, severe PE x mild PE. First, a univariate analysis was performed with each dependent variable in each model. Those parameters whose association with the dependent variable showed a significance ≤0.20 were included in multivariate analysis. The final model was set when all variables reached a significance value ≤0.05 and an adequacy measured by *Hosmer and Lemeshow* Test. All statistical tests were performed using a significance level of α = 0.05 and an adjusted significance level of α = 0.017 when *Bonferroni* correction was applied.

The sample calculation was performed based on the mean values for each parameter, which were obtained from similar studies including preeclamptic and normotensive pregnant women (mean ±SD) [12], [22], [23], considering: power  = 0.95; significance level  = 0.05 (OpenEpi v. 2, Rollins School of Public Health, Emory University, The United States of America. TGF-β1 was the factor that required a larger sample size (11 samples for both pregnancy groups) with a statistical power of 0.95 and 0.05 of confidence level.

## Results

### Demographic and clinical characteristics

No difference in age, body mass index and gestational age at blood sampling among groups was observed, while the gestational weigh gain and number of primiparous women were higher in the PE group (Table 1). As expected, preeclamptic women had significantly increased systolic and diastolic blood pressure compared with the two other groups (all *p*<0.001) (Table 1).

**Table 1:** Demographic and clinical characteristics of the three studied groups.

Variables	NP (n = 23)	Norm (n = 21)	PE (n = 43)	p value
Age (years) a	24 (23–29)	24 (19–27)	26 (22–30)	0.448^1^
BMI (Kg/m^2^)^b^	21.6±2.8	22.5±3.7	23.4±2.9	0.345^2^
GWG (Kg)^a^	N/A	10.2 (7.8–12.6)	13.5 (9.4–22.1)	0.024^1*^
GA at blood draw (weeks)^a^	N/A	35 (31–39)	34 (32–37)	0.667^1^
Primiparous (%)^c^	N/A	5 (24)	24 (56)	0.016^3*^
SBP (mmHg)^a^	110 (110–120)	110 (100–110)	160 (150–172)	<0.001^1**^
DBP (mmHg)^a^	70 (60–80)	70 (70–70)	107 (100–116)	<0.001^1**^

Abbreviations: BMI (body mass index: before pregnancy for normotensive pregnant women and preeclamptic women), GWG (gestational weight gain), GA (gestational age), SBP (systolic blood pressure), DPB (diastolic blood pressure), NP (non-pregnant women), Norm (normotensive pregnant women), PE (preeclamptic women), N/A (not applicable). Note: n =  total subjects.

a Data are presented as median (25^th^–75^th^ percentiles); ^b^Data are presented as mean ± standard deviation; ^c^Data are presented as number (percentage). ^1^Kruskal-Wallis with *Bonferroni* correction; ^2^ANOVA with *post-hoc* LSD;^ 3^Pearson chi-square (χ^2^) test. *p<0.05, **p<0.017.

Nineteen (44%) preeclamptic women were classified into early-onset PE and 24 (56%) into late-onset PE. Thirty-one (72%) PE women met the criteria for severe disease as defined by ACOG [1]. In the early-onset group, 90% manifested the severe form of the disease, in contrast with 58% women in the late-onset group. Two preeclamptic women with severe late-onset PE had features of the HELLP syndrome (hemolysis, elevated liver enzymes, low platelets).

### sEng, TGF-β1, sTNF-R1 and sTNF-R2

**Figure 1:**
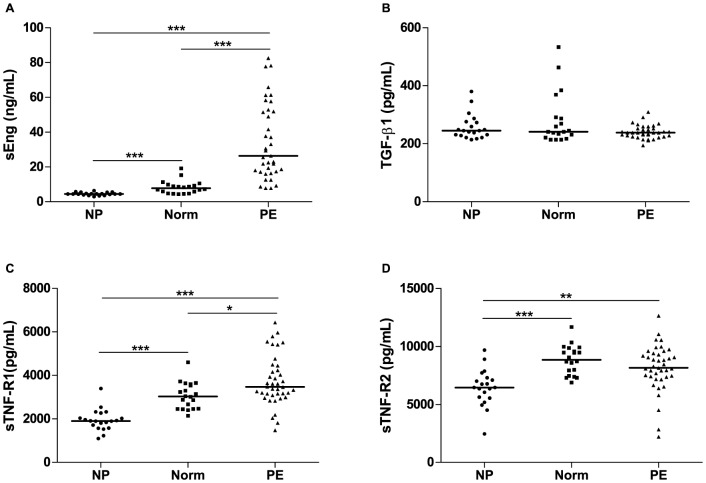
Plasma levels of sEng, TGF-β1, sTNF-R1 and sTNF-R2 in non-pregnant, normotensive pregnant and preeclamptic women. NP (non-pregnant women), Norm (normotensive pregnant women), PE (preeclamptic women). Horizontal bars represent median values for sEng, TGF-β1, sTNF-R1 and mean value for sTNF-R2. *p<0.05, **p<0.01 and ***p<0.001. Plasma levels of sEng (nanograms/milliliter) (A), sTNF-R1 (picograms/milliliter) (C) and sTNF-R2 (picograms/milliliter) (D) were higher in normotensive pregnant women than in non-pregnant women. When compared with normotensive pregnant women, sEng (A) and sTNF-R1 (C) were elevated in women with PE. TGF-β1 (picograms/milliliter) levels showed no significant differences among the studied groups (B).

Preeclamptic women showed increased plasma levels of sEng [29.34 (16.76–55.29) *vs.* 4.45 (3.94–5.10), *p*<0.001], sTNF-R1 [3479 (3182–4339) *vs.* 1909 (1649–2182), *p*<0.001] and sTNF-R2 [8698±1602 *vs.* 6508±1241, *p*<0.001] when compared with non pregnant women (Figures 1A, 1C and 1D). Plasma levels of sEng [29.34 (16.76–55.29) *vs.* 7.81 (4.78–9.42), *p*<0.001] and sTNF-R1 [3479 (3182–4339) *vs.* 3028 (2468–3606), *p* = 0.014] were also higher in preeclamptic comparing to normotensive pregnant women, but no difference was found for sTNF-R2 [8698±1602 *vs.* 8931±1277, *p* = 0.151] (Figures 1A, 1C and 1D). Moreover, normotensive pregnant women had higher sEng plasma levels [7.81 (4.78–9.42) *vs*. 4.45 (3.94–5.10), *p*<0.001], sTNF-R1 [3028 (2468–3606) *vs.* 1909 (1649–2182), *p*<0.001) and sTNF-R2 [8931±1277 *vs.* 6508±1241, *p*<0.001] comparing to non-pregnant women (Figures 1A, 1C and 1D). TGF-β1 plasma levels were similar among the three groups (*p* = 0.328) (Figure 1B).

**Figure 2:**
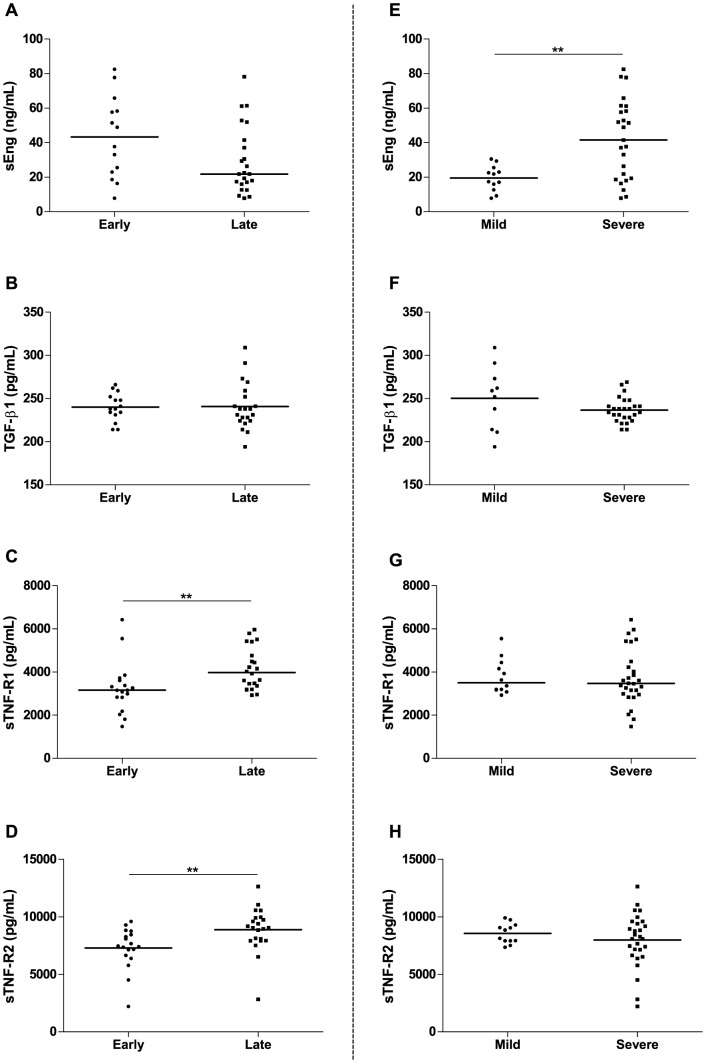
Plasma levels of sEng, TGF-β1, sTNF-R1 and sTNF-R2 according to the onset-time and severity of preeclampsia. Horizontal bars represent median values for sEng and sTNF-R1 and mean values for TGF-β1 and sTNF-R2. **p<0.01. Women with late preeclampsia (PE) had higher levels of sTNF-R1 (picograms/milliliter) (C) and sTNF-R2 (picograms/milliliter) (D) than women with early PE. No significant differences were found in sEng (nanograms/milliliter) (A) and TGF-β1 (picograms/milliliter) (B) comparing early and late PE. sEng levels were higher in severe PE than in mild PE (E) and no difference was found for TGF-β1 (F), sTNF-R1 (G) and sTNF-R2 levels (H).

We compared sEng, TGF-β1, sTNF-R1 and sTNF-R2 plasma levels according to the onset-time of PE (Figure 2, left panel). Late-onset PE showed higher levels of sTNF-R1 [3830 (3238–4693) *vs.* 3295 (2949–3638), *p* = 0.004] and sTNF-R2 [9249±1467 *vs.* 7673±1460, *p* = 0.003] comparing to early-onset PE (Figures 2C and 2D). Conversely, there was a tendency of increased sEng levels in early-onset comparing to late-onset PE [44.59 (21.29–60.19) *vs.* 22.19 (13.44–48.19), *p* = 0.077] (Figure 2A). There was also no significant difference in TGF-β1 plasma levels comparing the onset-time of the disease (Figure 2B).

We also compared sEng, TGF-β1, sTNF-R1 and sTNF-R2 plasma levels according to the severity of the disease (Figure 2, right panel). Only sEng plasma levels were significantly elevated in severe PE when compared to mild PE [44.59 (19.01–60.49) *vs.* 17.26 (11.74–24.53), *p* = 0.005] (Figure 2E).

### AST, ALT, LDH and creatinine plasma levels in PE women

AST, ALT, LDH and creatinine plasma levels were 27±14 U/L, 19 (9–27) U/L, 406 (336–522) U/L and 0.8±0.2 mg/dL, respectively. All the laboratory parameters were within the normal range (see methods section). These parameters were analyzed in PE according to the severity and onset-time of the disease. Only ALT levels were higher in severe PE [19 (8–21) U/L] than in mild PE [14 (9–25) U/L)] (*p* = 0.04), but these values were within the normal range. No other statistical differences were detected in mild PE *vs.* severe PE and early-onset PE *vs.* late-onset PE.

### Correlations between sEng, sTNF-R1, sTNF-R2 and clinical/laboratory parameters

**Figure 3:**
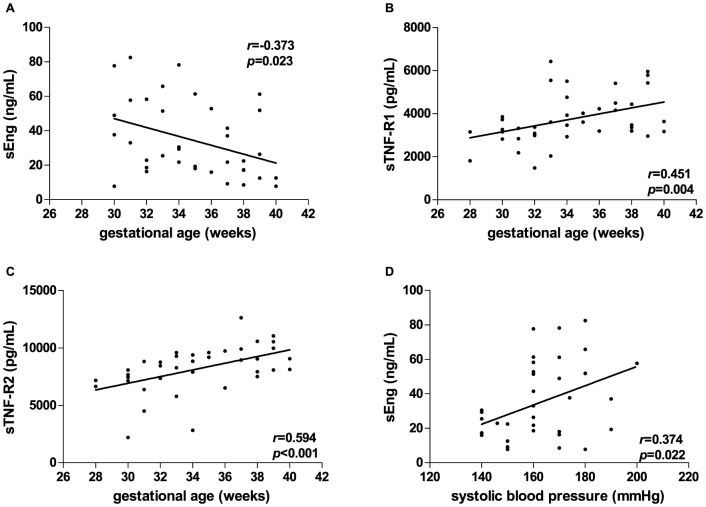
Significant correlations among sEng, TGF-β1, sTNF-R1 and sTNF-R2 levels and clinical parameters in preeclamptic women. The lines represent linear regression and the closed circles represent preeclamptic (PE) women. Maternal plasma sEng concentrations (nanograms/milliliter) are correlated negatively with gestational age (A), while a positive correlation was found between sTNF-R1 (picograms/milliliter) (B) and sTNF-R2 (picograms/milliliter) (C) plasma levels and gestational age in PE women. sEng plasma levels are also correlated positively with systolic blood pressure in PE women (D).

sEng levels showed a significant negative correlation with gestational age in PE (r = −0.373, *p* = 0.023) (Figure 3A) and a positive correlation with gestational age in normotensive pregnancy (r = 0.627, *p* = 0.003). In addition, sTNF-R1 (r = 0.451, *p* = 0.004) and sTNF-R2 (r = 0.594, *p*<0.001) levels increased according to the gestational age in PE group (Figures 3B and 3C). Moreover, sEng levels showed a positive correlation with systolic blood pressure in PE (r = 0.374, *p* = 0.022) (Figure 3D). There were no significant correlations between TGF-β1 levels and any clinical parameter in PE group.

**Figure 4:**
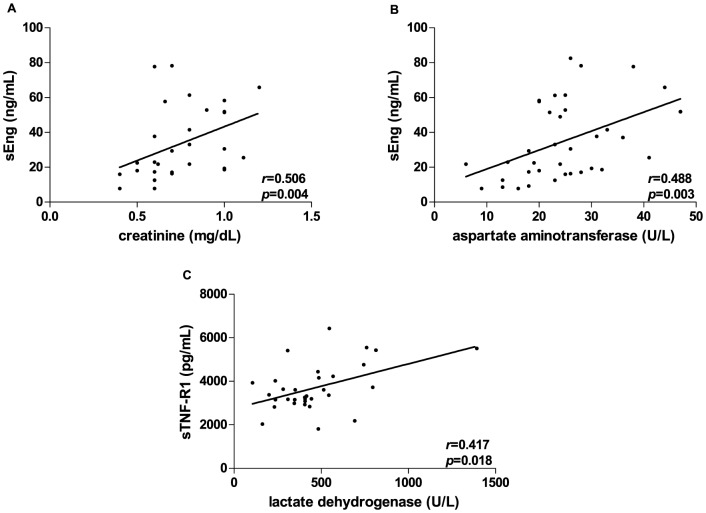
Significant correlations among sEng, TGF-β1, sTNF-R1 and sTNF-R2 levels and laboratorial parameters in preeclamptic women. The lines represent linear regression and the closed circles represent preeclamptic (PE) women. Maternal plasma sEng concentrations (nanograms/milliliter) are correlated positively with creatinine (mg/dL) (A) and aspartate aminotransferase (U/L) (B) levels in PE women. There is a positive correlation between sTNF-R1 (picograms/milliliter) and lactate dehydrogenase (U/L) levels in PE women (C).

In PE group, sEng levels were positively correlated with creatinine (r = 0.506, *p* = 0.004) (Figure 4A) and AST levels (0.488, *p* = 0.003) (Figure 4B), while sTNF-R1 and LDH levels were correlated positively (r = 0.417, *p* = 0.018) (Figure 4C).

sEng correlated positively with sTNF-R1 (r = 0.631, *p*<0.001) and sTNF-R2 (r = 0,378, *p* = 0.001) when all the participants of the study were included in the analysis. These correlations were not statistically significant after analyzing each group of pregnant women separately. As expected, sTNF-R1 and sTNF-R2 levels correlated positively with each other not only when all the study participants were included in the analysis (r = 0.575, *p*<0.001), but also in the groups of normotensive pregnant (r = 0.461, *p* = 0.041) and PE women (r = 0.497, *p* = 0.001). No significant correlation was detected between TGF-β1 levels and the other measured analytes.

### Multivariate logistic regression analyzes

sEng levels were independently associated with the disease severity (severe PE *vs.* mild PE: *p* = 0.014, β = 0.923, IC: 0.866-0.984) and pregnancy outcome (preeclamptic *vs.* normotensive pregnancy: *p* = 0.002, β = 0.669, IC: 0.522–0.859). Primiparity was also independently associated with pregnancy outcome (preeclamptic *vs.* normotensive pregnancy: *p* = 0.038, β = 0,091, IC: 0,009–0,871). No variable was independently associated with the onset-time of PE in multivariate logistic regression analysis.

## Discussion

In this study we compared the sEng, TGF-β1, sTNF-R1 and sTNFR-2 levels in women with different forms of PE, normotensive pregnant and healthy non-pregnant women.

The past decades of research have brought a greater understanding of potential angiogenic imbalance that is likely involved in the PE pathophysiology [24]. Eng is a pro-angiogenic agent that prevents apoptosis in hypoxic endothelial cells and is an essential component of the endothelial nitric oxide synthase (eNOS) activation complex, regulating nitric oxide-dependent regulation of vascular tone [25], [26]. In contrast, soluble Eng is an anti-angiogenic protein thought to impair TGF-β1 binding to its receptors and downstream signaling including effects on activation of eNOS and vasodilatation [7].

We found higher sEng levels in normotensive pregnant comparing with healthy non-pregnant women. sEng levels also increased with gestational age in normotensive pregnancy, indicating that the gestational age was associated with a greater anti-angiogenic profile. This finding is according to previous studies that found higher sEng and lower PlGF levels, and higher (sFlt1+sEng)/PlGF ratio in older gestational age [27], [28].

Previous studies have demonstrated that sEng is elevated in PE and before the onset of the disease [7]–[9]. In contrast to normotensive pregnant women, there is a negative association between sEng levels and gestational age in PE [29]. Accordingly, we found higher sEng levels in PE comparing to normotensive pregnant women that correlated negatively with gestational age. In addition, sEng levels were independently associated with the development of PE, reinforcing the role of sEng in disease pathogenesis. Our data demonstrated that primiparity was also independently associated with pregnancy outcome. In agreement with other studies [30], [31], we found a tendency of increased sEng levels in early-onset comparing to late-onset PE.

Consistent with previous reports [7], [30], we also found a significant increase of sEng levels in severe PE compared with mild PE. In addition, sEng levels were independently associated with the disease severity. It is well known that blood pressure and protein excretion are increased in preeclamptic women with the severe form of the disease [1]. A recent study has indicated an association between sEng and high blood pressure in pregnancy [32]. In one animal model of PE, the administration of adenovirus encoding sEng produced hypertension, proteinuria and endothelial dysfunction, further amplified by the coadministration of sEng and sFlt1, leading to a severe PE-like disease including HELLP syndrome and fetal growth restriction [7]. In line with these experimental data, we found that systolic blood pressure was positively associated with sEng levels in PE.

In accordance to Zhang *et al.* [33], we showed that sEng and creatinine levels were positively associated in PE. Angiogenesis play a critical role in renal homeostasis, since glomeruli form the functional barrier between the blood and urinary compartment. Genetic studies in mice have revealed an important role of the pro-angiogenic factors VEGF and PlGF in renal development and vascular health [34]. On the other hand, the anti-angiogenic factors sFlt-1 and sEng have been associated with glomerular damage and proteinuria. Venkatesha *et al*. [7] showed that proteinuria was modest in sEng-treated rats and severe in sFlt-1-treated group, while a nephrotic-range proteinuria was found using both sFlt-1 and sEng. Masuyama *et al.* [27] demonstrated a tendency of increased proteinuria levels in high sEng group compared to low sEng group.

Hepatic function may be significantly altered in PE women, especially in those with the severe form of the disease and this alteration is even more pronounced in HELLP syndrome [1]. Indeed, we found higher levels of ALT in severe PE than in mild PE, but these values were within the normal range. In this study, two patients with severe late-onset PE developed HELLP syndrome. ALT and AST levels were above the upper limit of the normal range in one of them. Our data also showed a positive correlation between sEng and AST levels in PE. In a study with rats treated with sEng or sFlt1 [7], an increase in AST levels was found, but no changes in liver histology was observed, while the group treated with sEng and sFlt1 showed even higher AST and areas of ischemia and necrosis. One recent study showed that sEng was positively correlated with peak liver enzyme levels in women with HELLP syndrome, but this correlation was not statistically significant and was also observed with sFlt-1 [22]. Taken these data together, it can be speculated that these two anti-angiogenic factors may contribute synergistically to the liver damage.

Circulating TGF-β1 levels in PE women compared with normotensive pregnant women have been previously studied but the data are not conclusive [12], [13]. In agreement with two reports [13], [35], our data showed similar TGF-β1 levels in PE and normotensive pregnant women. Some investigators, however, have found higher TGF-β1 levels in PE compared to normotensive pregnant women [12], [23], [36]. Increased sEng levels observed in PE women can impair TGF-β1 signaling in the vasculature by reducing the amount of TGF-β1 available to bind to its cell membrane receptors [7]. Moreover, platelet activation is a common feature in PE and activated platelets are the main sources of TGF-β1 in the circulation [37]. Thus, TGF-β1 secreted by activated platelets could play a compensatory role in preserving endothelial function in PE [23]. Data regarding TGF-β1 levels in normotensive pregnant compared with non-pregnant women are also conflicting. In agreement with other reports [35], [36], we found no difference in TGF-β1 levels between these groups. In contrast, Gil-Villa *et al.* [38] have reported higher levels of TGF-β1 in non-pregnant women, while Ayatollahi *et al.* [13] found the opposite. Several factors could affect circulating TGF-β1 levels in PE and normotensive pregnancy, such as ethnical and genetic background, body mass index and smoking. Differences in PE severity, as well as in gestational age of sampling might contribute to this controversy. Also, variations in procedures for obtaining and processing blood samples and in the techniques used for determining TGF-β1 could result in considerable variability in TGF-β1 levels [36]. In our study, both preeclamptic and normotensive pregnant women were in the third trimester of gestation at sampling and none of the studied pregnant women were overweight. Smoking was not included in the exclusion criteria of our study, because it was difficult to obtain accurate information about it. Moreover, we were unable to determine the women' ethnic groups due to the high genetic variability present in Brazilian population.

Maternal systemic inflammatory response is believed to play an important role in PE pathogenesis [15]. Normal third trimester pregnancy is a condition associated with enhanced systemic inflammation compared with the non-pregnant state, but less pronounced than in PE. Excessive release of pro-inflammatory cytokines, such as TNF-α, can disturb endothelial cell function, modulate cell adhesion molecules and induce the release of other pro-inflammatory cytokines [39]. Shedding of TNF-α soluble receptors from the cell membranes provide a mechanism for TNF-α activity inhibition. In addition, soluble TNF receptors are regarded as reliable markers of TNF-α activity as they have longer lives than their ligand, which allow them to circulate even in the absence of detectable free TNF-α [40].

Consistent with previous reports [41], [42], we show that both soluble receptors (sTNF-Rs) were elevated in normotensive pregnant women compared with non-pregnant women, which suggest that inflammatory responses are features of pregnancy itself. In accordance to Opsjøn *et al.* [43], we observed higher levels of sTNF-R1 and similar levels of sTNF-R2 in PE women compared with normotensive pregnant women. PE is associated with higher levels of TNF-α than normotensive pregnancy and it is believed that the TNF-R1 neutralizes TNF-α more efficiently than TNF-R2 [40], [44]. This hypothesis may explain why sTNF-R1 levels were increased in PE, while the levels of sTNF-R2 were similar between PE and normotensive pregnancy. Although there are differences in the efficiency to neutralize TNF-α, we observed a positive correlation between sTNF-R1 and sTNF-R2 in normotensive pregnant women and PE women, indicating that the levels of these factors simultaneously increase, even in the absence of disease.

In pregnancy, sTNF-Rs release in the circulation increases towards term [41]. Here, we showed a positive relationship between both soluble receptors levels and gestational age in PE. sTNF-Rs levels were not associated with PE severity. Together, these data support the higher TNF-Rs levels observed in the late-onset PE group compared with the early-onset PE group.

sTNF-R1 and LDH levels showed a positive correlation in PE women. Increased LDH levels can result from liver dysfunction and hemolysis, which occur especially in HELLP syndrome [45]. The patients with HELLP syndrome in our study had median levels of LDH above the upper limit of the normal range, while sTNF-R1 levels were above the 75^th^ percentile. Physiological induction of TNF-α is protective, but its overproduction seems to cause direct endothelial injury [46]. Furthermore, high levels of TNF-α are associated with lower expression of endothelial nitric oxide synthase and, consequently, reduced production of nitric oxide, which in addition to a vasodilator and a platelet inhibitor, has modulating action of leukocyte adhesion the endothelium [47], [48]. Indeed, liver of HELLP syndrome women display marked neutrophil infiltration and stain strongly with TNF-α and neutrophil elastase antibody [49]. Thus, extra-high levels of TNF-α could directly and indirectly harm endothelial liver cells and exacerbates the inflammatory response in this tissue, which, in turn, could contribute to enzyme elevation in HELLP syndrome. Despite LDH median levels in preeclamptic women are near the upper limit of the normal range considered in this study, we found no difference in LDH levels comparing mild and severe PE. sTNF-R1 median levels were also similar in mild and severe forms of the disease. These findings may explain why sTNF-R1 correlated with LDH but not with PE severity in our study.

Finally, sEng and sTNF-R1 levels were positively correlated in our study. Several lines of evidence support a role for transmembrane Eng and its soluble form (sEng) in diseases associated with exacerbated inflammatory responses, like PE. Eng is present in monocytes, and it is up-regulated during the monocyte-macrophage transition and in endothelial cells of inflamed tissues [50], [51]. Recent works support the role of Eng in leukocyte adhesion, transmigration and interleukin production [52], [53]. In opposite way, sEng has the ability to inhibit leukocyte adhesion [52]. Parrish *et al.* [54] showed that plasma and placental levels of sFlt-1, an antiangiogenic protein, increase in response to TNF-α induced hypertension in pregnant rats. Surprisingly, this effect was not observed for sEng.

In conclusion, our data suggest that excessive sEng and TNF-α release in the circulation may play an important role in PE pathophysiology, and that sEng is more associated with severe PE, while sTNF-Rs are more associated with late-onset disease. To date, this is the first study that evaluates sEng, TGF-β1, sTNF-R1 and sTNF-R2 plasma levels collectively in different clinical manifestations of PE. Large prospective studies are needed to determine whether these factors might yield predictive tools for PE prognosis.
